# A qualitative descriptive study of the group prenatal care experience: perceptions of women with low-risk pregnancies and their midwives

**DOI:** 10.1186/1471-2393-14-334

**Published:** 2014-09-26

**Authors:** Sarah D McDonald, Wendy Sword, Leyla E Eryuzlu, Anne B Biringer

**Affiliations:** Division of Maternal-Fetal Medicine, McMaster University, 1280 Main St. West, HSC 3N52B, Hamilton, Ontario L8S4K1 Canada; Departments of Obstetrics & Gynecology, McMaster University, 1280 Main St. West, HSC 3N52B, Hamilton, Ontario L8S4K1 Canada; Departments of Radiology, McMaster University, 1280 Main St. West, HSC 3N52B, Hamilton, Ontario L8S4K1 Canada; Departments of Clinical Epidemiology & Biostatistics, McMaster University, 1280 Main St. West, HSC 3N52B, Hamilton, Ontario L8S4K1 Canada; School of Nursing, McMaster University, 1280 Main St. West, Hamilton, Ontario L8S 4K1 Canada; Faculty of Health Sciences, McMaster University, 1280 Main St. West, Hamilton, Ontario L8S4K1 Canada; Department of Family and Community Medicine, University of Toronto, 60 Murray Street, Fourth floor, Toronto, Ontario M5T 3L9 Canada

**Keywords:** Group prenatal care, Pregnant women, Midwives, Satisfaction, Experience, Qualitative research

## Abstract

**Background:**

Group prenatal care (GPC) originated in 1994 as an innovative model of prenatal care delivery. In GPC, eight to twelve pregnant women of similar gestational age meet with a health care provider to receive their prenatal check-up and education in a group setting. GPC offers significant health benefits in comparison to traditional, one-on-one prenatal care. Women in GPC actively engage in their healthcare and experience a supportive network with one another. The purpose of this study was to better understand the GPC experience of women and care providers in a lower risk group of women than often has been previously studied.

**Methods:**

This qualitative descriptive study collected data through three focus group interviews - two with women who had completed GPC at a midwifery clinic in Ontario, Canada and one with the midwives at the clinic. Data was analyzed through open coding to identify themes.

**Results:**

Nine women and five midwives participated in the focus groups, from which eight categories as well as further subcategories were identified: The women and midwives noted reasons for participating (connections, education, efficiency). Participants suggested both benefits (learning from the group, normalizing the pregnancy experience, preparedness for labour and delivery, and improved relationships as all contributing to positive health outcomes) and concerns with GPC (e.g. sufficient time with the midwife) which generally diminished with experience. Suggestions for change focused on content, environment, partners, and access to the midwives. Challenges to providing GPC included scheduling and systems-level issues such as funding and regulation. Flexibility and commitment to the model facilitated it. Comparison with other models of care identified less of a relationship with the midwife, but more information received. In promoting GPC, women would emphasize the philosophy of care to other women and the midwives would promote the reduction in workload and women’s independence to colleagues.

**Conclusions:**

Overall, women and midwives expressed a high level of satisfaction with their GPC experience. This study gained insight into previously unexplored areas of the GPC experience, perceptions of processes that contribute to positive health outcomes, strategies to promote GPC and elements that enhance the feasibility of GPC.

## Background

Prenatal care was developed to reduce maternal morbidity and mortality [1], mainly from preeclampsia [2]. Later its goal was expanded to reduce low birth weight and preterm birth [1]. Today, prenatal care aims to improve the health of woman and their infants through the monitoring of maternal and fetal wellbeing, provision of health-related information and psychological support [3], monitoring, routine testing, early identification of risks or problems, and prevention [4].

The current traditional model of prenatal care comprises a longer first visit involving a complete history and examination, followed by an average of 13 short (10 to15 minutes), private visits with the clinician [2]. Prenatal care providers are experiencing pressure to see an increasing number of patients, resulting in patients receiving shorter appointments and longer wait times [5]. Due to the limited time with patients, answering questions and providing counseling on health behaviours is often limited [2].

Group care, initially conceived as a model of well-child care in 1974 [5], was developed for prenatal care, also known as CenteringPregnancy®, by Sharon Schindler Rising in 1994 [6]. Group prenatal care (GPC) was developed from Rising’s experience with family-centered approaches to prenatal care, as well as her recognition of the repetitious and inefficient nature of traditional one-on-one prenatal care [2]. GPC addresses the shortcomings of traditional prenatal care expressed by pregnant women and care providers; for instance, GPC provides longer visits, which allow for more in-depth discussions [5] and opportunities to gain experience, knowledge and skills in pregnancy [7]. In the GPC model, eight to twelve pregnant women of similar gestational age, often along with their partners or support persons, meet together with certified nurse midwives, certified midwives, nurse practitioners, or physicians for ten 90-minute prenatal visits that occur at regular time intervals throughout the pregnancy and culminate in a postpartum visit [3]. Similar to traditional care, the visits involve a standard prenatal risk assessment; however, pregnant women in GPC actively participate in their care and assessment, including measuring their own blood pressures and weights [3]. Risk assessments are followed by educational group discussions, and the opportunity for women to talk and share with one another, thereby offering social support [3]. Discussion topics often include healthy eating, pregnancy concerns and self-care, substance abuse, childbirth preparation, breastfeeding, contraception, and parenting strategies [2], with more opportunity for in-depth discussions [5].

The benefits of GPC in comparison to traditional prenatal care, noted by a systematic review, include lower rates of preterm birth (RR 0.71; 95% CI 0.52 to 0.96), fewer Caesarean sections (RR 0.80; 95% CI 0.67 to 0.93), and higher breastfeeding rates (RR 1.08; 95% CI 1.02 to 1.14) [8]. Other studies have found that women feel more ready for labour and satisfied with their care [9] in comparison to women in traditional care. Women who have participated in GPC have expressed satisfaction with: “getting more in one place at one time”; “[feeling] supported”; “learning and gaining meaningful information”; “not feeling alone in the experience”; “connecting”; “actively participating and taking ownership of care”; and overall “getting more than they realized they needed” [10]. Similarly, physicians involved in facilitating GPC appreciated: “sharing ownership of care”; “having a greater exchange of information”; learning more about the women and thus being more perceptive in their interaction with the women; “seeing women get to know and support each other”; “having more time”; “experiencing enjoyment and satisfaction in providing care”; and overall “providing richer care” [11].

GPC is currently implemented in over 300 settings in the United States, Canada, the United Kingdom, and Sweden [12]. However, there remains much room for further dissemination of the benefits and use of this model of care, including methods for promotion and improved accessibility to GPC.

The goal of this study was to gain a better understanding of women’s and care providers’ experiences with GPC in a setting where women have lower obstetrical risk than has usually been studied. The midwives in our study were in the early implementation stages of GPC, thus providing the opportunity to gain insight onto the reasons for choosing the GPC model, as well as challenges and successes. Hence, we explored beyond areas previously examined including certain aspects of the GPC experience, such as women’s and care providers’ motivation for participating in GPC, concerns about the model, perceived benefits of GPC, suggestions for change, challenges to providing GPC, and comparisons of GPC to other models of care [10, 13–17]. We sought to expand upon these areas particularly in the early phases of implementation of GPC in a midwifery clinic within a universal health care system and to understand other additional aspects of the experience not yet studied, such as perceptions of processes that contribute to positive health outcomes, strategies to promote GPC and elements that enhance the feasibility of GPC.

## Methods

### Design

A qualitative descriptive design was used to understand women’s and care providers’ experiences of participating in GPC. According to Sandelowski [18], qualitative descriptive is the method of choice when seeking to obtain straight descriptions of phenomena. This design provides a comprehensive summary of events in the everyday language of the participants (Sandelowski) [18].

### Setting

The study took place at a midwifery clinic in Ontario, Canada that had been offering GPC, in a format called Connecting Pregnancy, since August 2012. All participants in this study were involved in the GPC program offered at the clinic either as clients who were low-risk pregnant women (healthy and without anticipated complications) or midwife care providers. The study took place during April 2013, after the first few groups of women had given birth. Women were introduced to the idea of group prenatal care by the practice administrator during their first phone call to the clinic. When they had their first prenatal visit with their midwife, women were given further information about GPC and were asked about their interest in participating in this model of care.

Women who enrolled in GPC continued with their individual midwifery visits until mid-pregnancy, at which point the nine two-hour group sessions started. The sessions took place monthly up until the twenty-eighth week of pregnancy, when biweekly sessions began. Each group consisted of up to ten women of similar gestational ages and their partners. The beginning of each session involved two parts. After being taught how, women first measured and recorded their own weight and blood pressure using an automated blood pressure cuff, and tested a urine sample using a reagent strip to detect protein. Second, each woman had a brief, five-minute prenatal check-up with a midwife from her team. There were two midwifery teams at the clinic, and typically there was a midwife from each team present at the beginning of the sessions for the check-up. This provided an opportunity for each woman to meet with one of the midwives on her own midwifery team. Overall, each session lasted for two hours: a half hour for measurements and check-up; and the remaining time in the group space for discussion, self-care activities (e.g., dipping urine), and videos, facilitated by one midwife. Each session had a different focus topic that aligned with the women’s stage of pregnancy: the first was nutrition; the second was exercise and included a visit from a personal trainer; the third focused on emotions and mental health, and how to prepare for the baby and the postpartum period; sessions four, five and six were about labour, including information on vaginal births, comfort measures, and complications; the seventh was about the immediate postpartum period, such as infant medications and newborn screening; the eighth discussed breastfeeding; and the ninth session was a less-structured opportunity for a final gathering. If a woman had a preterm baby, she often brought her baby and her stories to the ninth session to share with the group.

### Recruitment and sampling

Women and midwives at a midwifery clinic in Ontario, Canada were recruited in April 2013. All women and midwives who were English-speaking and had completed GPC were invited to participate. A purposeful criterion sampling approach was used [19] choosing these groups because of the characteristics they represented in terms of the early implementation stages of GPC.

### Data collection

Data were collected using focus groups that lasted between 60 and 90 minutes. The focus groups were held in April, 2013, at the location of the GPC. Two of the focus groups consisted of women who were enrolled in GPC and the third focus group was with the midwives who led the GPC program.

The focus groups were conducted by two trained research staff using a semi-structured interview guide. Research staff provided a description of the purpose of the study and obtained written informed consent from participants prior to beginning data collection. Participants had the right to withdraw at any point during the focus group.

The open-ended questions in this guide sought to understand women’s and care providers’ motivators for participating in GPC, such as their willingness to enrol, aspects of GPC that appealed and did not appeal to them, their perceived advantages of the model and of processes that contributed to positive health outcomes, strategies to promote GPC and elements that enhanced the feasibility of GPC. Some questions asked specifically about their thoughts on the structure of GPC, such as the leadership of the group, the make-up of the women participating with regards to similarities and differences in their characteristics, the inclusion or exclusion of women’s partners, and the scheduling and duration of the sessions. All focus group interviews were digitally recorded and transcribed verbatim. During transcription, participants were assigned a number sequentially in the order in which they spoke, except for when the specific individual who was speaking was not discernable, in which case the comment was labelled “unidentifiable”. The study was approved by the Hamilton Health Sciences/McMaster University Faculty of Health Sciences Research Ethics Board.

### Data analysis

NVivo 8 software was used to organize the qualitative data. Transcripts were read in their entirety and then analyzed using open coding to assign conceptual codes to meaningful sections of the data. The research staff who performed the coding met with the principal investigator to confirm the coding scheme to be used across all three focus group interview transcripts, and data with common meanings were coded identically through comparative analysis. Pattern coding, which captured high-frequency codes, was used to identify specific dimensions of the GPC experience. Patterns in the data informed the identification of eight categories that reflected the GPC experience. Subcategories were identified for five of the categories to capture their specific elements.

## Results

In total, nine women and five midwives participated in the focus groups, which were undertaken within two months of the completion of the first few GPC groups. These women were drawn from a larger study of GPC (unpublished data) in which the participants had a mean age of 30 years, and the majority of whom were first time mothers (78%), self-identified as Caucasian (90%), had a combined household income of at least 60,000 CDN dollars (90%), and were either married or in common-law relationships (100%).

The eight categories under which the findings are organized are listed in Table 1. Although the women and the midwives participated in separate focus groups, their overall responses and impressions of GPC were very similar. Therefore their responses were grouped together when presenting the results. Exemplary quotes are used to illustrate themes. The sources of the quotes are identified with either W (woman) or MW (midwife) and their study participant number. In the case of the women, the number of the focus group is included as well.

**Table 1:** **Categories and subcategories**

Categories	Subcategories
1. REASONS FOR CHOOSING TO PARTICIPATE IN GPC	Connecting and networking
	Education and preparation
	Time and efficiency
2. BENEFITS PROVIDED BY GPC	Making connections
	Learning from the group
	Normalizing the pregnancy experience
	Improved relationships between women and midwives
	Feeling prepared for labour and delivery
	Reduction in workload
	Shift in social support
3. CONCERNS ABOUT GPC	
4. SUGGESTIONS FOR CHANGE	Content and process
	Physical environment
	Access to own midwifery team
	Presence of partners
	Participation of midwifery student
5. CHALLENGES TO PROVIDING GPC	Scheduling difficulties
	System-level challenges
6. FACILITATORS TO PROVIDING GPC	Flexibility of midwives
	Midwives’ commitment to GPC
7. COMPARISON WITH OTHER MODELS OF PRENATAL CARE	
8. SUGGESTIONS FOR PROMOTING GPC	

### Reasons for choosing to participate in group prenatal care

The women and midwives provided several reasons for choosing the group model of care. These included: connecting and networking; education and preparation; and time and efficiency.

#### Connecting and networking

The women stated that the opportunity to connect and share their pregnancy experiences with other women was an important part of their decision to participate in GPC. One woman explained, “*We had only lived in [region] for like a couple months…so I didn’t know any other women and so it seemed like a great opportunity to actually meet some other people.*” (FG1-W2) Another woman discussed how a social network can help overcome isolation:

*That sort of isolation of being on your own and trying to sort things out was kind of difficult, especially if you don’t have a big network of other moms at that time…I think that having people who are at the same stage as you is really important.* (FG1-W5)

Expanding on the importance of social connections, the women emphasized their desire for connections with “*like-minded*” women, such as other women who also preferred the midwifery philosophy of care. One woman reflected, “*I think it was nice…to be surrounded by like-minded people who are also seeking out that same approach…I find that it’s harder to relate to other people if…what you’re looking for is so different than what they are.*” (FG1-W5)

The women also hoped the connections would continue into the postpartum period in order to have a community of new mothers and babies at the same stage. “*I was really excited to know that…you could connect with people in pregnancy and then especially afterwards because I think that having people who are at the same stage as you is really important.”* (FG1-W5)

The midwives discussed a number of factors that informed their decision to offer GPC. The primary motivation was to provide a community for the pregnant women to connect with one another. They viewed these relationships to be very important. As one midwife commented:

*We really noticed, as midwives, that people were looking for something and they wanted to be connected to a group of women, and that’s really lacking right now, in our day and age, and this gives them the opportunity to find women that they have things in common with.* (MW3)

Another midwife explained, “*I think we’ve all experienced isolation as…one of the biggest barriers new mothers and new parents face*”. (MW1)

#### Education and preparation

The women chose to join GPC, in part, for the opportunity to learn from other pregnant women and their partners. They hoped to learn not only from hearing answers to their questions but also from the questions of others. One woman shared, *“You don’t really know what’s normal…and what’s something a little bit outside of that…So, being in a group was good for me in terms of other people articulating their questions.”* (FG1-W1) Another woman commented, “*My husband and I…didn’t really know what to ask, so we thought…everybody else…might have questions that we wouldn’t think of*.” (FG2-W4)

The women also were attracted to GPC by the extent of education they would receive. They liked how prenatal class content was included in the model so that they would not have to go elsewhere or pay for classes. One woman stated, “*[I liked] getting the prenatal classes and your prenatal care all wrapped together…you didn’t have to seek out education elsewhere because you were getting it as part of your prenatal care here”.* (FG2-W4) Women found the education to be more detailed than what they would have received elsewhere. Another woman commented: *“It seemed to be more in-depth than regular education…you get to find out exactly what was going to happen to you…step-by-step…it was important for me to know as much as possible in advance”.* (FG2-W1)

Similarly, the midwives hoped that GPC would provide better prenatal and postnatal education along with labour and postpartum preparation for the women, as illustrated by this comment: *“They get sort of the free childbirth classes content as well… They’ve just got…a bigger knowledge base and more confidence”.* (MW1)

#### Time and efficiency

Several of the women expressed how the longer time spent with the midwives (two-hour group sessions instead of half-hour individual appointments) was another factor in deciding to attend GPC. One woman remarked, *“It was kind of nice to be able to spend that extra time, even though it was in a group, to sort of get to know the person that will be performing such a personal service for you”.* (FG2-W3)

The midwives did not comment on the extra time spent with the women in the group model of care. However, they hoped that the GPC model would be a more efficient approach to providing prenatal care. One midwife commented, “*I think we were hoping that it would cut down on clinic time. It would be a more efficient way to run our clinics*”. (MW1)

### Benefits of providing group prenatal care

A number of benefits for women were discussed by both the women and the midwives. These included: making connections; learning from the group; normalizing the pregnancy experience; improved relationships between women and midwives; and feeling prepared for labour and delivery. In addition to recognizing the benefits to the women, the midwives identified other benefits including the reduction in workload and the shift in social dependency.

#### Making connections

The social connections and support from the group were identified as being beneficial to both the women and their partners. One woman reflected on the support she received from other women in GPC and the midwives who led the group:

*I think that the time and care that you’re given is wonderful. You feel really well supported, like everyone has said, both from your midwife team but also from the other people that you connect with. And I just think it helps you feel, you know … well support, I guess, is the best word.* (FG1-W5)

GPC was enjoyed for its social aspects. As one woman said, “*We have a really …fun time…but it’s not like fun-fun. You know what I mean? It’s something valuable fun.*” (FG1-W4) Another woman explained, *“It made it more fun too actually, because sometimes you might be really tired and then someone’s husband would have energy and just be making jokes, and I’d think ‘Oh, that’s nice. I needed some levity”.* (FG2-W2)

The midwives described the connections and the community built among the women as a benefit of the program, especially for women who were experiencing isolation outside of their care. One midwife described how *“[the women] are getting chattier as the sessions continue…exchanging emails and forming a tight community and just talking, where they would never do that in the waiting room*”. (MW3)

#### Learning from the group

The opportunity to learn from other group members was a benefit identified by both the women and the midwives. The women believed they benefitted from the experiences shared by the second or third time parents in the group. One woman explained that hearing the information first-hand from the other women also helped her husband:

*We ended up having a home birth…I hadn’t really thought of it until like a few weeks prior and then he was okay with it too, and I don’t think he would have been without the group sessions and hearing the stories.* (FG1-W3)

The women commented that they were more mindful about eating well and exercising because of GPC. One woman stated that the group helped her be healthier through peer pressure: “*I felt that there was like almost a classic kind of peer pressure component to it that helped me be more healthy.*” (FG1-W2) The women also appreciated getting useful information about exercise from the other group members, such as suggestions about exercise programs and where to access them. As one woman stated, “*Having other people give suggestions on what they were doing to basically stay active was really, really nice…stuff that you might not have thought about.*” (FG2-W4)

The midwives believed that some women gave more credibility to information that came from other women than that provided by midwives alone. One midwife explained:

*Authority from peers kind of trumps our authority, right? I could tell her something ‘til I’m blue in the face, but if they have somebody that they’re sitting next to and chatting with, somehow that’s more valid ‘cause it’s real life*. (MW3)

#### Normalizing the pregnancy experience

The women and midwives described how hearing other group members talk about their pregnancy experiences helped the women normalize the common discomforts of pregnancy and provided them with a different perspective. Knowing that others shared their experiences helped to reduce anxiety, which one woman believed could produce better health outcomes for their babies. The woman explained, “*And knowing that I wasn’t the only one going through it made me feel a lot more relaxed…I think that was probably good for the baby too – me being more relaxed*”. (FG2-W2) From the group discussions, the women gained new perspectives on what is normal and what should cause more concern. One woman reflected:

*When you hear other women say, ‘Oh no, that’s not happened to me’…it gives me a bit of freedom to allow myself to acknowledge the things that were really difficult. Or the other way: if you’re feeling like something’s really tough and then you hear someone else is having a much more difficult time, it kind of gives you some perspective on, ‘Okay, well this is a part of being pregnant.’* (FG1-W1)

One midwife explained, “*It really reassures them that what they’re going through is the normal process and that other women are experiencing it as well”.* (MW4)

#### Improved relationships between women and midwives

Both the women and the midwives discussed the impact GPC had on improving the relationship between the women and their midwives. The program’s structure provided the women with more opportunities to get to know and interact with the midwives at the clinic. As a result, the women were more likely to know the midwife who would deliver their baby, reducing their anxiety about the possibility of their own midwife not being available for their delivery. One woman explained, “*The midwife who ended up delivering for me was not one of my two team members originally…but she…was the co-facilitator of my group so I knew her…so that was good for me.*” (FG2-W2) The women also believed that the longer sessions allowed them to spend more time with their midwives and interact with them in a more informal context. One woman commented, “*You really get a chance to know them and they got to know you better as well because you were spending two hours with them instead of just, you know, the half hour session.*” (FG1-W2) Another woman explained, “*I think it was very positive just getting to know them in a very informal environment…you could really see how midwives interacted with everyone else.*” (FG2-W2) One midwife commented on the improved relationship: “*I don’t have any women looking at the calendar saying ‘O my gosh! When are you off-call?’ which happened all the time in the other model we worked in”.* (MW Unidentifiable)

#### Feeling prepared for labour and delivery

The women described being prepared and confident for labour and the postpartum period as a result of the program. They believed that their partner also felt better prepared, as conveyed in this remark:

*I think for [my partner] it helped him help me through the pregnancy, and I think when it came to the delivery time, we both commented on…how much more prepared we felt, even though you still don’t know what to expect ‘cause it’s a new and unexpected thing, we both just felt so much more relaxed and prepared.* (FG1-W1)

For the women whose deliveries did not go as planned, they believed they had acquired the information and the preparation needed to make good decisions during their delivery. One woman commented:

*The last prenatal group classes I was at was the one about complications, so about like inductions and having your care transferred and c-sections. Actually, I was prepared. Like, I had the information and knew all about like when intervention escalates other interventions.* (FG1-W1)

#### Reduction in workload

The midwives reported that GPC helped reduce their workload. Rather than discussing pregnancy topics with each client, the midwives were able to present the information once in the group setting. The midwives also noted that they were no longer seen as the only “experts” in that the pregnant women began to turn to each other for information and learning, which reduced the need for individual attention by the midwives. One midwife explained, “*We’ve had a huge reduction in post-partum clinic visits with people coming in…groups”.* (MW2)

#### Shift in social support

The midwives perceived a benefit in a shift in women’s social dependency from the midwives and to the women in the group, which was seen to be advantageous for both the midwives and the women. The women in the program had a larger support network available for providing support and information, compared to the two-person midwife team in the traditional individual model of care. This allowed the midwives to focus on their clinical role and allowed the women to relinquish some of their social dependency on their midwife. One midwife described the shift:

*I think taking away some of the pressure from us… We’ve all had clients who really want to latch on to you and be your best friend. Taking some of that pressure off and saying, ‘You know what? There’s a whole group of women here who are kind of at the same stage of life as you, who you can connect to’. It takes a little bit of the pressure off of us as well to be kind of all things to everybody. To be their midwife and their best friend and their mother…it maybe defines our clinical role a little more clearly in some respects and takes away from some of that social role.* (MW2)

Another midwife commented, “*[The pregnant women] don’t have the same attachment to us, which is healthy…there isn’t that same dependence or tie to the individual midwife, which is a good thing.*” (MW4) One midwife stated, “*I think that information is getting through and it is empowering them in a different way.*” (MW3) Another midwife described a card she got from one of the new mothers that acknowledged this change:

*Coming to group helped me be such a better mother.’ And I just thought, isn’t that so much better than saying, ‘You were wonderful. You were amazing. I couldn’t have done it without…’ ‘I am a better mother.’ And it was all her. It shifts the focus from us to them, and that’s what it should be about.* (MW1)

### Concerns about group prenatal care

Neither the women nor the midwives had any major reservations about participating in GPC, but they did identify some minor concerns. Some of the women were initially concerned about not having enough individual time with a midwife or the opportunity to ask questions in private; this was no longer a concern once the program began. The women had the opportunity to meet individually with a midwife at the beginning and end of each session, and those who wished were invited to book an individual appointment as well. One woman remarked, “*They were very clear that I was always welcome to schedule an individual appointment at any time that I felt that I needed one…and I didn’t end up doing that.*” (FG1-W5) One of the midwives was concerned about her lack of facilitation or group skills: “*I love the model; I really want to do this but…I’m not a trained or a skilled facilitator and so there’s that sort of uncertainty about, ‘How’s this going to go…is it going to work for me?*” (MW1)

### Suggestions for change

Overall, the women were satisfied with their GPC experience, as exemplified by this comment: “*I was very satisfied with the group, and…I would do it again for our next [pregnancy].*” (FG1-W2) The midwives described GPC as an evolving process and were satisfied with its progression to date. One midwife stated, “*I’m very satisfied. It’s hard work but good work, and I think we’re seeing the rewards of doing it.*” (MW3)

When asked if there were any aspects of the model they would like to change, the women and midwives made a few suggestions for improvement in regards to the content and process, the physical environment, and the access to their own midwifery team. Although not phrased as concerns, the women raised two issues that they perceived to have had an impact on the groups: the presence of partners and the participation of student midwives.

#### Content and process

A few women said they would prefer more information specifically on pain management, labour and delivery, such as the birthing process and coping methods. One woman said, “*I almost a took separate pain management course offered by another practitioner, but it was full. But it was fine but…I was thinking at the end, ‘Oh, I wish I had gotten more of that.*’” (FG2-W1)

The presentation style differed according to the midwife that led the group session. The women shared that they would have preferred more group facilitation and opportunities for discussion instead of a lecture style of presenting information. One woman reflected, *“I was expecting…more group sharing and more discussion, and the format didn’t quite meet my expectations.*” (FG1-W2) This was also an area identified by the midwives to be improved upon; the midwives commented that while they are skilled in their practice, group facilitation is a completely separate skill that requires some additional training.

The midwives expressed a desire to have more professionals and specialists, such as nurses, nutritionists, or physiotherapists, visit the group and give presentations. They felt that this would result in a more collaborative model of care, and would reduce the burden on them. They anticipated a challenge in obtaining funds to pay the guest speakers because of the midwifery funding model.

#### Physical environment

Both the women and midwives thought the physical space was sometimes less than ideal. This occurred when enrolment exceeded expectations and the available space was inadequate. The timing of the group sessions likely had an impact on the adequacy of space, with more preferable times being more likely to be over-crowded, as conveyed by one woman: “*One thing I found was that because we had a large group, because we were at the good time slot, and most of the dads came, I found that the room got very hot…there wasn’t always enough space”.* (W Unidentifiable) One midwife explained, “*Twelve women with their partners is a very, very full room.*” (MW Unidentifiable)

#### Access to own midwifery team

A few of the women thought that there was limited time to get to know their own midwifery team. Some of the women said they took control to ensure they were more familiar with their own midwives. One woman explained:

*[The midwife] was not on my midwifery team but she did our group sessions. So, when I scheduled [my individual appointments] at the end – thirty-seven, thirty-eight, thirty-nine weeks…I specifically asked to meet with the people who were on the team to deliver.* (FG1-W5)

It was suggested that the format of the program be modified to eliminate this problem. One woman commented:

*I think that even just having more midwives available during the belly checks and then maybe you could ensure that you really got to know the people on your team if they were here during the belly checks because that was really only in the first half hour… I think that that was definitely something that they attempted to put in place. So, maybe working towards doing that would be helpful.* (FG1-W5)

However, other women stated that they were able to see each midwife on their team equally during the group session and assessments. As another woman said, *“I found I was able to see all the midwives equally*”. (FG1-W3)

The midwives tried to ensure that most women were able to get to know the midwives on their own teams. They explained how the physical assessments and individual appointments were with a member of the woman’s midwife team rather than with another clinic midwife.

#### Presence of partners

The women discussed the impact that partner involvement had on the group sessions. Some mentioned that the presence of male partners might have changed the dynamics of the group, but not necessarily in a negative way. One woman suggested:

*I don’t think it was negative…I think it was good to have the partners there because I think there are a lot of advantages to them being there, but you’re right, I think it would have been different as well to have just women.* (FG1-W5)

Others did not explicitly state that the male partners should not attend, but they did believe there was an impact from their presence. Another woman described the potential impact as follows:

*Sometimes I did feel that having men in the room kind of made me feel a little bit less willing to share some of the more intimate details of the pregnancy…there are times like when we started getting into like things like constipation and stuff that I felt a little bit shy to like really be too open about what I was experiencing.* (FG1-W2)

It was suggested that perhaps partners could attend specific sessions designed to be of benefit to them, with the remaining sessions reserved for the pregnant women only.

#### Participation of midwifery student

While acknowledging that the student midwife who facilitated the group sessions was skilled, several women shared that they would have preferred to have a more experienced midwife in this role. One woman explained, *“I kind of wish in the beginning it was the midwife and then the student would sort of gradually be introduced.*” (FG2-W3) Another woman stated:

*The student was great and she was there for my birth and she was amazing and very nice…but…I wanted to hear answers from somebody that I felt had more experience ‘cause I had none, and it was important to me to get that experience aspect.* (FG2-W1)

### Challenges to providing group prenatal care

A few challenges to the GPC model were identified by the women and midwives. These consisted of scheduling difficulties and system-level challenges.

#### Scheduling difficulties

For some women, the GPC session dates and times most appropriate for their delivery date were not convenient for themselves or their partners. Some opted to participate in a group that was more convenient for them, even though their delivery date did not match as closely with the other women. It was suggested that more evening sessions should be available.

The midwives reported that the scheduling of the prenatal groups was very challenging. They acknowledged that the assistance of their administrator was essential to overcoming the challenge of balancing the schedules of the midwives and clients. One midwife noted, “*The scheduling is a nightmare. So, I think your administrator has to be a hundred percent on board.*” (MW1)

The midwives also acknowledged that the timing of the group sessions was difficult for some women, yet the predictability of the schedule was helpful for others. One midwife remarked, “*The scheduling is rigid, so there have been people who have been really interested in group but not able to sign up because they don’t have that flexibility to just be available every time.*” (MW1) “*But then that’s an advantage for other women. That it’s predictable,*” another midwife explained (MW4).

#### System-level challenges

The midwives explained how the Ontario funding formula for midwifery care provided a financial challenge to offering GPC. Midwives are paid when a woman is discharged from their care. The challenge in the GPC model presents itself when midwives facilitate a group for women who are not on their designated caseload. The funding formula was also one of the difficulties associated with paying other health care professionals to contribute to the program through guest presentations. One midwife explained:

*But if our funding formula was more flexible…that would certainly help…maybe a bit more similar to the B.C. model where you, as a midwife, you bill the Ministry of Health directly, and you get a course of care fee or maybe several times during a pregnancy and postpartum you get paid rather than…We get paid one lump sum. It comes through a transfer payment agency and then to the clinic, and a portion of that is kept by the clinic for admin work.* (MW2)

The regulatory College of Midwives of Ontario restricts the number of midwives involved in a woman’s care to a maximum of four. This can pose a barrier to GPC as midwifery clinics might divide up their staff into teams of more than four. One midwife reflected:

*The college requirements…around continuity of care as well can be a challenge if you can have no more than four midwives named on a chart. And…there are five of us here. What if that’s a fifth name on the chart?* (MW1)

### Facilitators to providing group prenatal care

The midwives identified two qualities that they perceived to facilitate the implementation of GPC: their flexibility and their commitment to the GPC program.

#### Flexibility of midwives

The midwives believed that their flexibility and ability to adjust to last-minute changes was a contributing factor to the success of the program. They needed to be prepared and able to step in to cover a group session if, for example, the midwife scheduled to facilitate was attending a birth. One midwife explained, “*Midwives are pretty good at being flexible and changing plans at the last minute.*” (MW4)

#### Midwives’ commitment to group prenatal care

The midwives held a strong commitment to the program and trusted each other, which they believed facilitated implementation of the program. A midwife commented, “*If we weren’t committed to this, it would have been easy to walk away*”. (MW4) The midwives discussed the equal contribution of each midwife to GPC and their efforts to all teach and facilitate in a consistent manner across all groups. Another midwife explained that there were no feelings of competitiveness among her colleagues. No one would say, “*‘I want to create my best group so everybody will want to be in, you know, MW2’s group…’ We’re all working together and delivering together…putting that individual ego thing aside and saying this is a group process.*” (MW2)

### Comparison with other models of prenatal care

Some women compared GPC to their experiences with other models of care. They found that GPC provided them with more time with their care provider and with more information without having to ask for it first. One woman remarked:

*My appointments with my [obstetrician] were under ten minutes in length and very quick…I had to ask a lot of information and this way you’re given a lot of information. And I didn’t have to like pull it out of someone. It was just given to me, which is nice because you don’t always know the questions to ask.* (FG1-W5)

One of the women thought she did not get to know her midwife as well, but she did not view this as a negative aspect of GPC. She explained:

*Two children, both under the care of midwives. I really found, for all the reasons that we’ve talked about, it was wonderful to connect with other people. I maybe felt I knew my midwife a lot better the first time around…And, as I said, this time there was lots of different people. So, that was different, but not necessarily better or worse.* (FG1-W5)

When comparing GPC to the way in which they had previously practiced, the midwives also commented that they did not get to know their clients as well in the GPC model as they did in the traditional midwifery model.

### Suggestions for promoting group prenatal care

With regards to promoting GPC, the women primarily commented on emphasizing the philosophy of care, which is generally similar to the midwifery philosophy. One woman suggested providing details about the benefits of the program: “*So, part of the promotion being, ‘These are the benefits of taking part in this kind of a group,’ and being more explicit about that.*” (FG1-W1) It also was recommended that the benefits of networking and being part of a community of pregnant women be emphasized in promoting GPC.

The midwives discussed ways in which to promote the program to other midwives, such as by highlighting the women’s increased independence and the ways in which women benefit from sharing information and learning from each other, thus further reducing the need for individual attention by the midwives. The midwives also suggested promoting the reduction in workload, along with the reduction in clinic visits, in-between visit contacts with clients, and postpartum contacts. One midwife explained that, with GPC, there are “*reduced pages, reduced anxiety, reduced visits in your clinic schedule.*” (MW Unidentifiable)

## Discussion

Our study furthers the understanding of women’s and care providers’ experiences of GPC by describing motivators, benefits, concerns, suggestions for change, challenges, and comparisons to other models of prenatal care in a recently established GPC setting. Unique to our study are the novel insights on the women’s and midwives experience with GPC, perceptions of processes that contributed to positive health outcomes, strategies to promote GPC and elements that enhanced the feasibility of GPC.

Our study identified several novel features of the GPC experience for the women. Women expressed a benefit from the positive peer pressure of the group, which they thought contributed to improved health behaviours, such as exercising. Some women initially were concerned about not having enough individual time with their midwife, but this concern usually resolved as the GPC experience progressed. To improve GPC, the women suggested providing more information on pain management and access to their own midwifery team. With regards to promoting GPC, the women recommended emphasizing the philosophy of care, the longer time spent with the care provider, and the network and community built for the pregnant women.

Novel insights into the midwives’ GPC experience also were obtained, including a reduction in their workload. They suggested involving more professionals and specialists in the program to create a more collaborative model of care. The midwives identified facilitators of GPC: the flexibility of the midwives at this centre and their commitment to GPC. The midwives noted several barriers to providing GPC: adequate physical space, scheduling, the provincial midwifery funding formula, and regulatory issues, some shared by previous literature [13].

Some of our other findings are supported by previous studies describing women’s experiences in GPC. Similar to the women in our study, Kennedy et al. identified women’s concerns with not having enough private time with their care provider [15]. Other studies have found similar benefits of GPC, such as reduction in feelings of isolation [17] and helping women not feel like “the only one” [10]. Additionally, other studies have reported normalization of social and economic situations [17] and of pregnancy experiences [10, 13, 15–17], with reduction in anxiety [17]. Previous literature also has noted the potential beneficial health impacts garnered by women from GPC, such as greater control or empowerment [13] and involvement in their healthcare [10, 15, 17]. GPC was reported to be a more efficient model of prenatal care in other studies [10], and women have found the two-hour sessions to be “enough time” [17] and to have felt accommodated if they wished for extra visits [16]. The presence of partners, while valued by some, may have inhibited others [16]. GPC is seen as a “fun” model of care which, once women have experienced, they would “overwhelmingly” opt for over individual care for subsequent pregnancies [17].

Previous studies described similar findings to our study on several aspects of the care provider experience, including women sharing information amongst themselves [11] and more active participation in their own care [11]. Midwives reported perceived benefits in the connections built for themselves, student midwives, and pregnant women and their partners [20]. In previous studies, midwives were initially concerned with their group facilitation skills, but they gained in confidence with time and found that their “facilitation was getting better” [13].

In addition to the novel aspects of our study, another strength is that we captured the perspectives of both women and midwives who had participated in GPC. Furthermore, the women participating in our study had low-risk pregnancies whereas participants in most other qualitative studies of GPC were high-risk women, thereby increasing our understanding of the GPC experience among a low-risk, more generalizable population.

Our findings are limited in that the participants in our focus groups were from one GPC location. The women in our study voluntarily chose to enrol in GPC, which may have predisposed them towards having more favorable perceptions of the model. As well, our study did not capture the experience of partners attending GPC, but only the perceived impact on the partners described by the women. Furthermore, our study had a relatively small sample size and member checking was not conducted. Achieving saturation was not an objective of the sampling approach.

Care providers who are interested in implementing GPC may benefit from the novel findings we have obtained on ways in which to promote GPC to both women and care providers while maintaining flexibility and commitment to GPC. Our study supports the need for future research into the feasibility of overcoming the system-level challenges to GPC expressed by the midwives. Furthermore, there is a need to identify methods of enhancing midwives’ facilitation skills to better support the group model of learning. Implications of this and other work, including the systematic reviews which show improved perinatal outcomes [8], should include broader support for GPC, within and outside of midwifery, including reducing barriers such as regulations and funding issues.

## Conclusion

This qualitative descriptive study adds further support to the benefits of group prenatal care experienced by both low-risk women and their care providers. Women in GPC are able to network with other pregnant women and build better relations with their midwives in a way that provides them with greater knowledge, satisfaction, confidence, and support. Our study has gathered novel input from women and midwives regarding facilitators and ideas for promoting GPC, all of which may contribute to the continued development and expansion of this model, with the ultimate goal of improving prenatal care and the overall health of women and their infants.

## Authors’ information

SDM, MD, MSc (Clinical Epidemiology) is a Maternal-Fetal Medicine specialist (high risk obstetrician), Clinician-Scientist and an Associate Professor in the Department of Obstetrics & Gynecology and is an Associate Member in the Department of Clinical Epidemiology and Biostatistics and Diagnostic Imaging at McMaster University. As a Clinician-Scientist her salary is supported by a Canadian Institute of Health Research New Investigator Award. Her research in perinatal epidemiology focuses on weight-related issues during pregnancy and the postpartum period, and on related health services.

WS is a Professor and Assistant Dean (Research) in the School of Nursing, and an Associate Member of the Department of Clinical Epidemiology and Biostatistics, Faculty of Health Sciences, McMaster University.

AB is an Associate Professor of Family and Community Medicine at the University of Toronto, and holds the Ada Slaight and the Slaight Family Foundation Directorship in Maternity Care for the Ray D. Wolfe Department of Family Medicine, Mount Sinai Hospital, in Toronto.

## Abbreviation

GPC: Group prenatal care.

## References

[1] Alexander GR, Kotelchuck M (2001). Assessing the role and effectiveness of prenatal care: history, challenges, and directions for future research. Public Health Rep.

[2] Novick G (2004). CenteringPregnancy and the current state of prenatal care. J Midwifery Womens Health.

[3] Rising SS (1998). Centering pregnancy. An interdisciplinary model of empowerment. J Nurse Midwifery.

[4] Kirkham C, Harris S, Grzybowski S (2005). Evidence-based prenatal care: Part I. General prenatal care and counseling issues. Am Fam Physician.

[5] Walker DS, Worrell R (2008). Promoting healthy pregnancies through perinatal groups: a comparison of CenteringPregnancy(R) group prenatal care and childbirth education classes. J Perinat Educ.

[6] Centering Healthcare Institute: *The Centering Model*. Accessed at http://www.centeringhealthcare.org/pages/about/history.php on March 31, 2014. 2014. Ref Type: Online Source

[7] Massey Z, Rising SS, Ickovics J (2006). CenteringPregnancy group prenatal care: promoting relationship-centered care. J Obstet Gynecol Neonatal Nurs.

[8] Ruiz-Mirazo E, Lopez-Yarto M, McDonald SD (2012). Group prenatal care versus individual prenatal care: a systematic review and meta-analyses. J Obstet Gynaecol Can.

[9] Ickovics JR, Kershaw TS, Westdahl C, Magriples U, Massey Z, Reynolds H, Rising SS (2007). Group prenatal care and perinatal outcomes: a randomized controlled trial. Obstet Gynecol.

[10] McNeil DA, Vekved M, Dolan SM, Siever J, Horn S, Tough SC (2012). Getting more than they realized they needed: a qualitative study of women’s experience of group prenatal care. BMC Pregnancy Childbirth.

[11] McNeil DA, Vekved M, Dolan SM, Siever J, Horn S, Tough SC (2013). A qualitative study of the experience of CenteringPregnancy group prenatal care for physicians. BMC Pregnancy Childbirth.

[12] Shields SG, Candib LM (2010). Women-Centered Care in Pregnancy and Childbirth.

[13] Baldwin K, Phillips G (2011). Voices along the journey: midwives’ perceptions of implementing the CenteringPregnancy model of prenatal care. J Perinat Educ.

[14] Fereday J, Collins C, Turnbull D, Pincombe J, Oster C (2009). An evaluation of midwifery group practice. Part II: women’s satisfaction. Women Birth.

[15] Kennedy HP, Farrell T, Paden R, Hill S, Jolivet R, Willetts J, Rising SS (2009). “I wasn’t alone”–a study of group prenatal care in the military. J Midwifery Womens Health.

[16] Andersson E, Christensson K, Hildingsson I (2012). Parents’ experiences and perceptions of group-based antenatal care in four clinics in Sweden. Midwifery.

[17] Novick G, Sadler LS, Kennedy HP, Cohen SS, Groce NE, Knafl KA (2011). Women’s experience of group prenatal care. Qual Health Res.

[18] Sandelowski M (2000). Whatever happened to qualitative description?. Res Nurs Health.

[19] Patton MQ (2002). Qualitative Research & Evaluation Methods (3rd Edition).

[20] Teate A, Leap N, Homer CS (2013). Midwives’ experiences of becoming CenteringPregnancy facilitators: a pilot study in Sydney, Australia. Women Birth.

[CR21] The pre-publication history for this paper can be accessed here:http://www.biomedcentral.com/1471-2393/14/334/prepub

